# Soil Enzyme Activities and Microbial Nutrient Limitation Between Restored Vegetation and Primary Forest on the Loess Plateau

**DOI:** 10.3390/plants15203113

**Published:** 2026-10-12

**Authors:** Juanjuan Cui, Zixuan Wang, Sishan Zou, Sibei Chen, Huijie Xiao

**Affiliations:** School of Soil and Water Conservation, Beijing Forestry University, Beijing 100083, China; cui02100281@126.com (J.C.); wzx2243505@bjfu.edu.cn (Z.W.); zs34688@bjfu.edu.cn (S.Z.); sbc0811@outlook.com (S.C.)

**Keywords:** soil enzyme activity, microbial metabolism, stoichiometric homeostasis, vegetation restoration, Loess Plateau

## Abstract

Vegetation restoration aims to recover ecosystem functions in degraded landscapes, yet differences in soil microbial functioning between restored vegetation and primary forests remain poorly understood. In this study, we investigated extracellular enzyme activities (EEAs) and ecoenzymatic stoichiometry in plantation, secondary grassland, and primary forest on the Loess Plateau and evaluated the relative contributions of multiple environmental factors to their variations. The results showed that grassland exhibited higher EEAs than plantation and primary forest. Vector lengths (1.34–1.38) indicated greater microbial C limitation in plantation than in grassland and primary forest, whereas vector angles (40.60–43.21°) indicated predominantly greater N than P limitation across vegetation types. Soil organic carbon, slope gradient, and alkaline phosphatase were the main predictors of EEAs, microbial C limitation, and microbial N/P limitation, respectively. In addition, microbial biomass stoichiometric homeostasis varied among vegetation types, with comparatively weak homeostasis in plantation and strict homeostasis in grassland. Overall, grassland was characterized by high enzyme investment and relatively low microbial nutrient limitation, suggesting that natural secondary succession following cropland abandonment may support favorable soil microbial functioning on the Loess Plateau.

## 1. Introduction

Soil extracellular enzymes catalyze the depolymerization of organic matter and regulate the accessibility of carbon (C), nitrogen (N), and phosphorus (P) to plants and microorganisms [1,2]. Critical enzymes include β-1,4-glucosidase (BG), β-1,4-N-acetylglucosaminidase (NAG), L-leucine aminopeptidase (LAP), and alkaline phosphatase (ALP), with BG, NAG + LAP, and ALP frequently used as proxies for microbial investment in C-, N-, and P-acquiring processes, respectively [3,4]. Moreover, Cellulase (CE) contributes to cellulose degradation, whereas polyphenol oxidase (PO) is an oxidative enzyme associated with phenolic and other relatively recalcitrant C compounds [5,6]. Catalase (CA) decomposes hydrogen peroxide and is commonly treated as an indicator of soil oxidative stress and microbial activity [7]. Because extracellular enzyme production reflects microbial resource-acquisition strategies, ecoenzymatic stoichiometry integrates the relative allocation of C-, N-, and P-acquiring enzymes and can be used to evaluate microbial responses to resource imbalance [8,9,10,11]. Vector analysis further integrates these stoichiometric relationships to quantify relative microbial C limitation and N versus P limitation [3]. Therefore, combining soil extracellular enzyme activities (EEAs) with ecoenzymatic stoichiometry and vector analysis provides a complementary framework for assessing microbial resource acquisition and nutrient limitation under different vegetation conditions [12].

Primary forest is generally characterized by stable nutrient cycling, high biodiversity, and strong ecosystem resilience, making it an important reference ecosystem for evaluating the ecological effects of vegetation restoration [13]. Under increasing anthropogenic disturbance and environmental change, large areas of primary forest have degraded, while plantation and secondary grassland have become widespread vegetation types during subsequent restoration and natural succession [14]. These changes in vegetation cover substantially alter plant community composition, soil nutrient availability, and microbial community structure, thereby reshaping soil microbial metabolic processes [15]. Although numerous studies have reported changes in EEAs following vegetation restoration, the observed responses remain inconsistent. Some studies have reported higher EEAs in primary forest, potentially associated with greater soil nutrient availability and moisture [16,17,18], whereas others have found higher EEAs in restored vegetation, possibly reflecting increased microbial investment in enzyme production under resource limitation [19]. These contrasting responses may be attributed to variations in environmental factors [20]. For example, tree species can influence EEAs by altering litter quality and root characteristics [21], whereas soil nutrient status, substrate availability, and aggregates can regulate enzyme production, kinetics, and catalytic efficiency [22,23]. Topographic conditions can affect EEAs by modifying the redistribution and availability of soil nutrients and moisture [24]. In addition, soil microorganisms may respond to changes in resource availability by regulating their biomass elemental composition [4]. Microbial biomass stoichiometric homeostasis reflects the extent to which microbial C:N:P composition is maintained or adjusted in response to variation in soil resource stoichiometry and therefore provides a complementary perspective on microbial adaptation to resource fluctuations [3].

Dryland ecosystems are particularly sensitive to vegetation restoration due to water limitation, soil erosion, and nutrient constraints [19]. The Chinese Loess Plateau provides a representative system for examining these interactions because it has experienced extensive conversion of primary forest to agricultural land, resulting in severe soil erosion and land degradation [25]. Since the 1990s, the implementation of the “Grain for Green” Project has promoted the establishment of extensive plantations, substantially advancing vegetation restoration [4]. Meanwhile, cropland abandonment represents another important pathway for vegetation restoration, facilitating the natural development of grassland [26]. Numerous studies have investigated soil EEAs and their ecoenzymatic stoichiometry across different vegetation types on the Loess Plateau [3,4,27]. Nevertheless, their findings remain inconsistent, which has been attributed to differences in environmental conditions, such as soil properties, vegetation characteristics, and microbial communities [26,28]. Recently, several studies have further suggested that these contrasting responses may partly reflect fundamental differences between restored vegetation and primary forest [29]. Although studies have reported substantial differences in plant species composition, soil nutrient conditions, and microbial community structure between restored vegetation and primary forest [30,31], it remains unclear whether different restoration pathways lead to distinct soil microbial functional responses. In particular, how microbial resource acquisition, nutrient limitation, and stoichiometric regulation differ between active afforestation through plantation establishment and natural succession following cropland abandonment, and how these responses compare with those in primary forest, remain poorly understood. 

In our study, we aimed to determine the variations in soil microbial resource-acquisition and stoichiometric strategies between restored vegetation and primary forest on the Loess Plateau. The specific objectives were (1) to determine the variations in EEAs and ecoenzymatic stoichiometry among different vegetation types; (2) to determine the variations in microbial biomass stoichiometric homeostasis among different vegetation types; and (3) to identify the environmental factors associated with variations in EEAs and microbial nutrient limitation. We hypothesized that (1) EEAs and microbial nutrient limitation differed among vegetation types due to differences in resource availability and that (2) microbial biomass stoichiometric homeostasis differed among vegetation types, reflecting contrasting microbial strategies for coping with variation in soil resources.

## 2. Results

### 2.1. Soil EEAs in Different Vegetation Types

**Figure 1:**
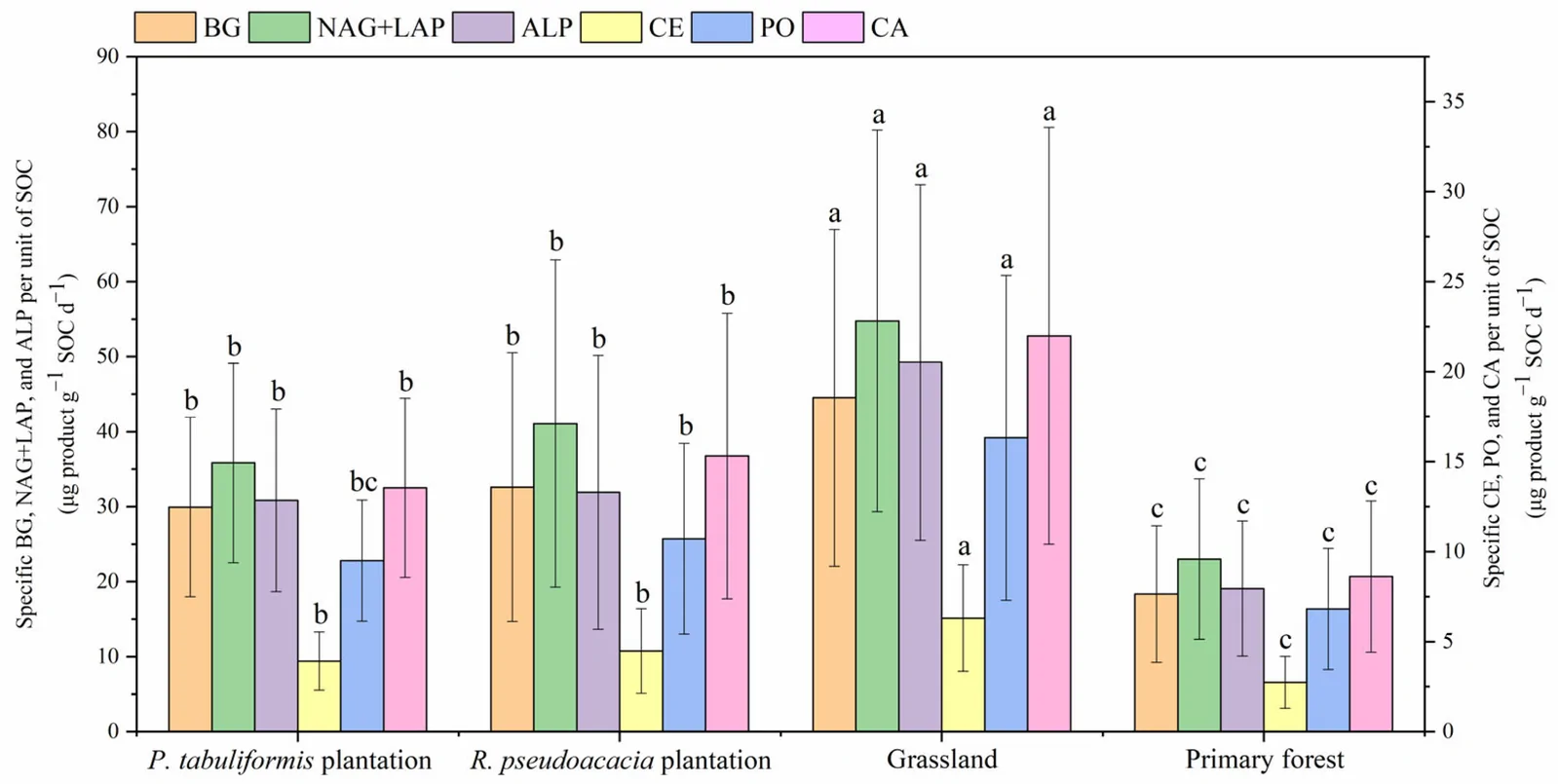
Soil enzyme activities of different vegetation types. BG, β-1, 4-glucosidase; NAG, β-1, 4-N-acetylglucosaminidase; LAP, L-leucine aminopeptidase; ALP, alkaline phosphatase; CE, cellulase; PO, polyphenol oxidase; CA, catalase. Different lowercase letters indicate that the differences between different vegetation types are significant (*p* < 0.05). The values are expressed as mean ± SD (error bar).

Grassland exhibited higher EEAs than plantation and primary forest, with mean BG, (NAG+LAP), ALP, CE, PO, and CA activities of 44.53 μg glucose g^−1^ SOC d^−1^, 54.77 μg NH_4_^+^-N g^−1^ SOC d^−1^, 49.23 μg phenol g^−1^ SOC d^−1^, 6.31 μg glucose g^−1^ SOC d^−1^, 16.33 μg purpurogallin g^−1^ SOC d^−1^, and 22.00 mL KMnO_4_ g^−1^ SOC d^−1^, respectively (*p* < 0.05) (Figure 1). In contrast, primary forest generally exhibited the lowest EEAs, with corresponding mean values of 18.35 μg glucose g^−1^ SOC d^−1^, 23.02 μg NH_4_^+^-N g^−1^ SOC d^−1^, 19.09 μg phenol g^−1^ SOC d^−1^, 2.74 μg glucose g^−1^ SOC d^−1^, 6.82 μg purpurogallin g^−1^ SOC d^−1^, and 8.62 mL KMnO_4_ g^−1^ SOC d^−1^, respectively. For PO, primary forest had lower activity than the *R. pseudoacacia* plantation (*p* < 0.05), but did not differ from the *P. tabuliformis* plantation (*p* > 0.05). No significant differences in EEAs were detected between the two plantation types (*p* > 0.05).

### 2.2. Relationships Between EEAs and Soil Properties

**Figure 2:**
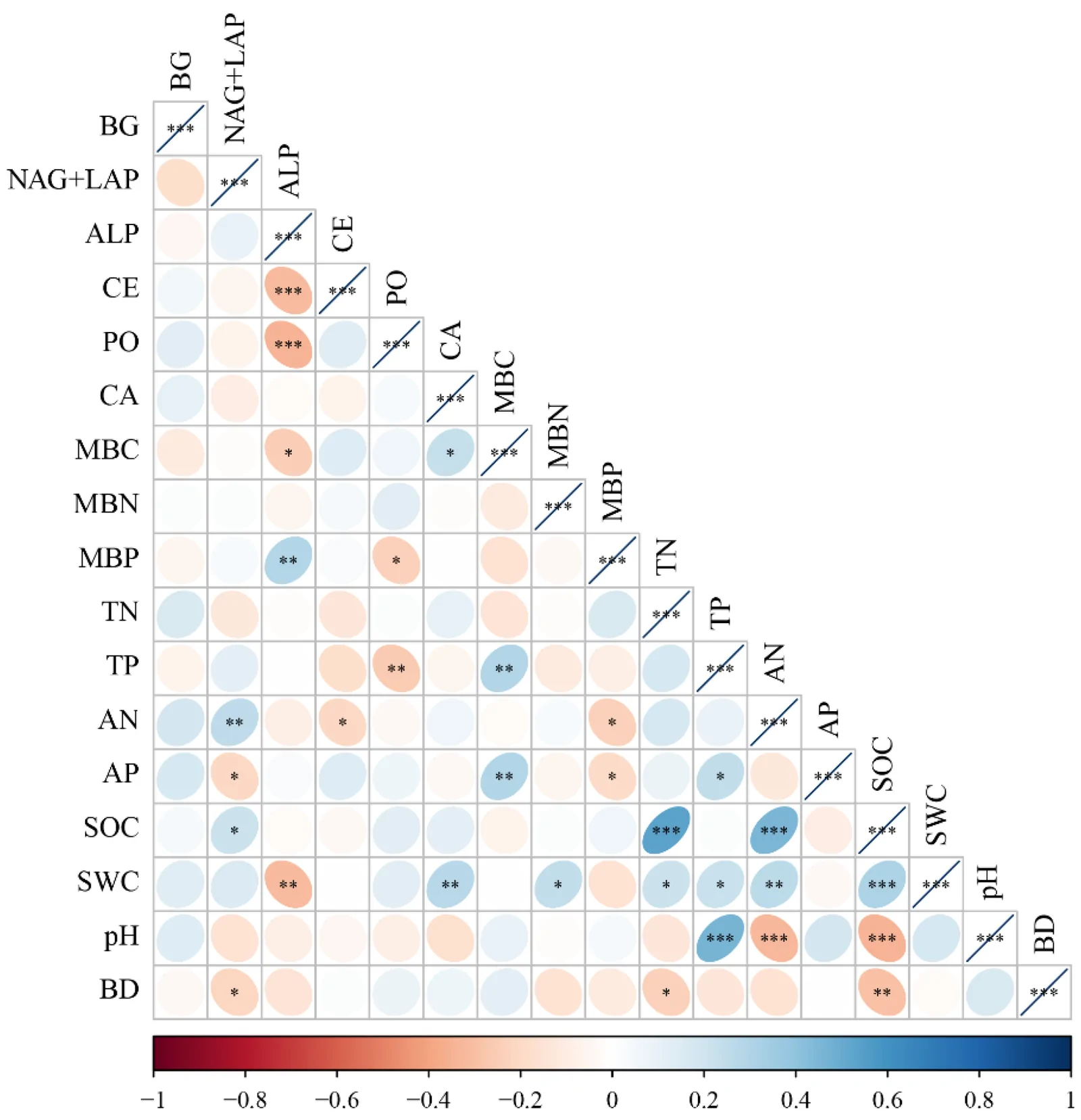
Correlation matrix analysis of different soil enzyme activities. Blue indicates positive correlations, while red indicates negative correlations. “*” indicates that the correlation is significant (*p* < 0.05). “**” indicates that the correlation is highly significant (*p* < 0.01). “***” indicates that the correlation is extremely significant (*p* < 0.001). The eccentricity of the ellipse represents the strength of the correlation. BG, β-1, 4-glucosidase; NAG, β-1, 4-N-acetylglucosaminidase; LAP, L-leucine aminopeptidase; ALP, alkaline phosphatase; CE, cellulase; PO, polyphenol oxidase; CA, catalase; MBC, microbial biomass carbon; MBN, microbial biomass nitrogen; MBP, microbial biomass phosphorus; AP, available phosphorus; AN, available nitrogen; TP, total phosphorus; TN, total nitrogen; SOC, soil organic carbon; SWC, soil water content; BD, bulk density.

ALP was negatively correlated with CE and PO (*p* < 0.001), while the correlations among the other EEAs were nonsignificant (*p* > 0.05) (Figure 2). In addition, (NAG + LAP) was positively correlated with SOC and AN (*p* < 0.05) but negatively correlated with AP and BD (*p* < 0.05). ALP was positively correlated with MBP (*p* < 0.05) but negatively correlated with MBC and SWC (*p* < 0.05). CE was negatively correlated with AN (*p* < 0.05). PO was negatively correlated with MBP and TP (*p* < 0.05). CA was positively correlated with MBC and SWC (*p* < 0.05). No significant correlations were detected between BG and soil properties (*p* > 0.05).

### 2.3. PCA of EEAs and Soil Properties

**Figure 3:**
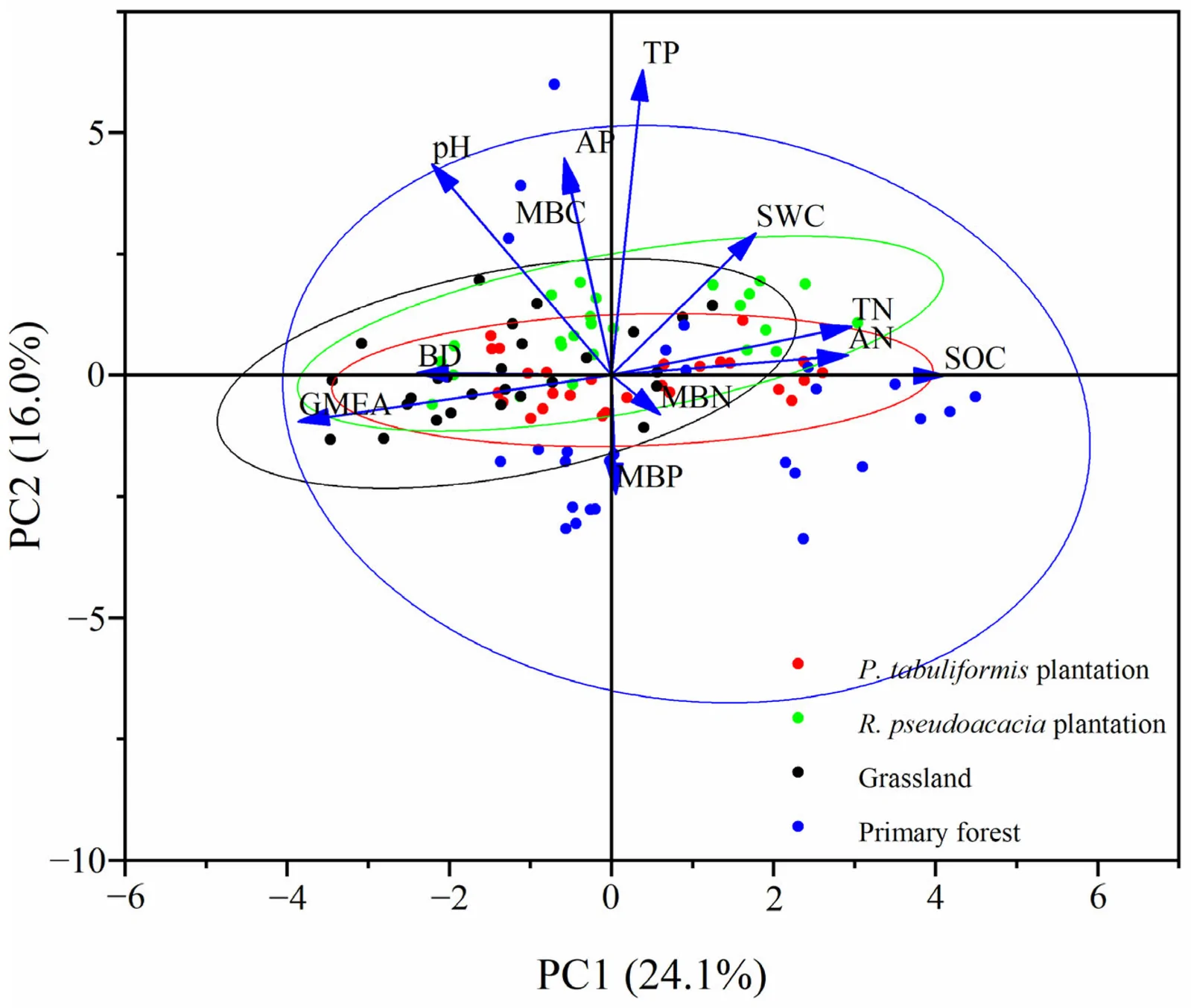
Principal component analysis (PCA) of soil enzyme activities and soil properties in different vegetation types. GMEA, geometric mean of enzyme activity; MBC, microbial biomass carbon; MBN, microbial biomass nitrogen; MBP, microbial biomass phosphorus; AP, available phosphorus; AN, available nitrogen; TP, total phosphorus; TN, total nitrogen; SOC, soil organic carbon; SWC, soil water content; BD, bulk density.

The PCA indicated that EEAs and soil properties significantly changed among different vegetation types (Figure 3). The contribution rates for PC1 and PC2 were 24.1% and 16.0%, respectively. Specifically, PC1 was positively associated with SOC, AN, TN, SWC, and MBN and negatively associated with GMEA and BD, while PC2 was positively associated with MBC, TP, AP, and pH and negatively associated with MBP. In comparison to other vegetation types, primary forest was associated with higher soil nutrients, while grassland was associated with higher GMEA. With respect to plantations, *P. tabuliformis* plantation was associated with higher SOC, AN, MBN, and MBP, while *R. pseudoacacia* plantation was associated with higher TN, SWC, TP, AP, MBC, pH, BD, and GMEA. In addition, our study showed more scattered distributions in plots of primary forest, which indicated that primary forest had comparatively higher variability in EEAs and soil properties among individual plots than plantation and grassland.

### 2.4. Vector Analysis of Microbial Nutrient Limitation

**Figure 4:**
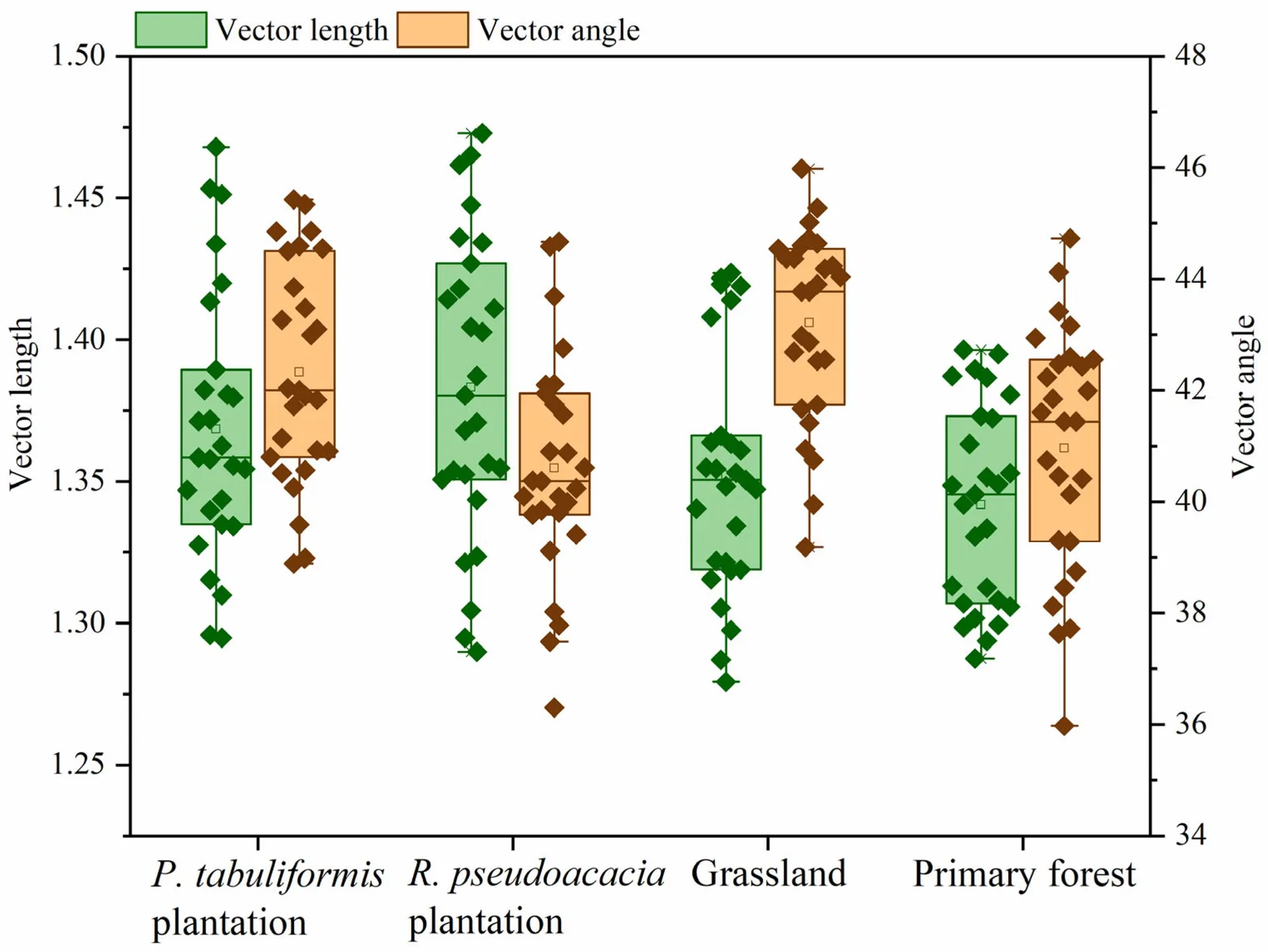
Microbial nutrient limitation of different vegetation types based on vector analysis. The ends of the boxes represent the 25th and 75th percentiles. The central lines and empty squares represent the medians and means, respectively. The maximum whisker length is 1.5 times the interquartile range.

In our study, the vector length varied across different vegetation types (Figure 4). Specifically, *R. pseudoacacia* plantation had the highest mean vector length, with a value of 1.38, while primary forest had the lowest mean vector length, with a value of 1.34. Therefore, the microbial carbon limitation was highest in *R. pseudoacacia* plantation and lowest in primary forest. In addition, the mean vector angles ranged from 40.60° to 43.21° among different vegetation types, which reflected microbial N limitation. However, we also found vector angles exceeding 45° in *P. tabuliformis* plantation and grassland, suggesting potential microbial P limitation within their soil microorganisms.

### 2.5. Stoichiometric Homeostasis

**Figure 5:**
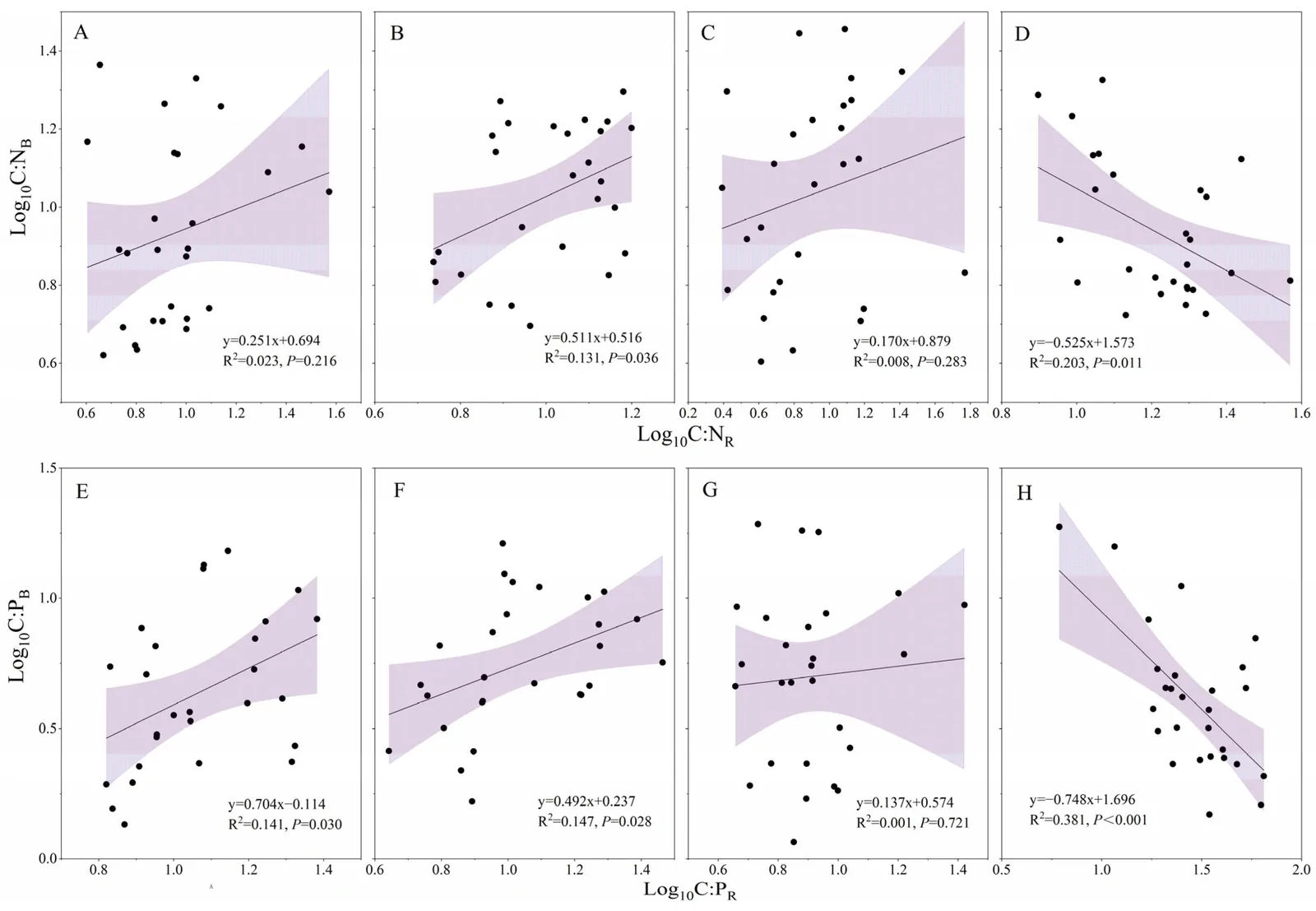
Soil microbial biomass homeostasis related to N and P acquisition in *Pinus tabuliformis* plantation (**A**,**E**), *Robinia pseudoacacia* plantation (**B**,**F**), grassland (**C**,**G**), and primary forest (**D**,**H**). log_10_C:N_B_ and log_10_C:P_B_ represent the base-10 logarithm of microbial biomass C:N and C:P ratios, respectively. log_10_C:N_R_ and log_10_C:P_R_ represent the base-10 logarithm of soil resource C:N and C:P ratios, respectively. The shaded areas represent the 95% confidence intervals of the fitted regression lines.

Our study determined the strength of stoichiometry homeostasis by analyzing the associations between microbial biomass stoichiometry ratios (C:N_B_ and C:P_B_) and soil resource stoichiometry ratios (C:N_R_ and C:P_R_) (Figure 5). The log_10_C:N_B_ was significantly correlated with log_10_C:N_R_ in *R. pseudoacacia* plantation and primary forest, with slopes of 0.511 and −0.525, respectively (*p* < 0.05) (Figure 5B,D). Based on the prespecified positive-slope classification, *R. pseudoacacia* plantation was weakly plastic; the negative primary forest slope fell outside the classification range and was therefore not assigned a homeostasis class. No significant correlations were detected between log_10_C:N_B_ and log_10_C:N_R_ in *P. tabuliformis* plantation and grassland (*p* > 0.05), which indicated that the strength of the stoichiometry homeostasis was strictly homeostatic (Figure 5A,C).

log_10_C:P_B_ was significantly correlated with log_10_C:P_R_ in *P. tabuliformis* plantation, *R. pseudoacacia* plantation, and primary forest, with slopes of 0.704, 0.492, and −0.748, respectively (*p* < 0.05) (Figure 5E,F,H). Based on the prespecified classification, *P. tabuliformis* plantation was weakly plastic, while *R. pseudoacacia* plantation was weakly homeostatic. The negative primary forest slope fell outside the classification range and was therefore not assigned a homeostasis class. No significant correlations were detected between log_10_C:P_B_ and log_10_C:P_R_ in grassland (*p* > 0.05), which indicated that the strength of the stoichiometry homeostasis was strictly homeostatic (Figure 5G).

### 2.6. Factors Affecting EEAs and Microbial Nutrient Limitation

**Figure 6:**
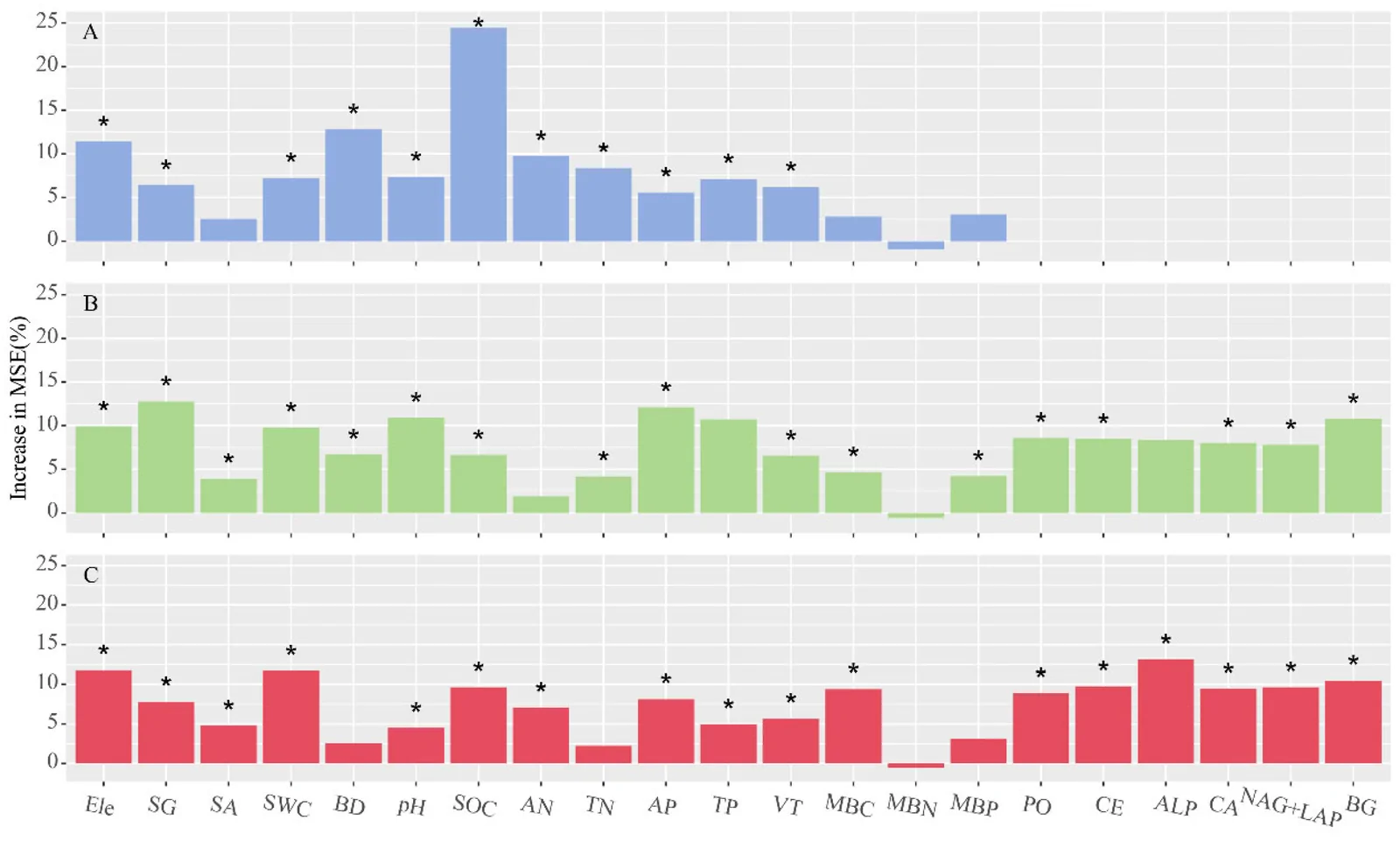
Effects of different driving factors on the variations in (**A**) GMEA, (**B**) vector length, and (**C**) vector angle. The importance of different driving factors was evaluated using the percentage increase in the mean squared error (%IncMSE) of the random forest model. * *p* < 0.05. GMEA, geometric mean of enzyme activity; MBC, microbial biomass carbon; MBN, microbial biomass nitrogen; MBP, microbial biomass phosphorus; AP, available phosphorus; AN, available nitrogen; TP, total phosphorus; TN, total nitrogen; SOC, soil organic carbon; SWC, soil water content; BD, bulk density; Ele, elevation; SG, slope gradient; SA, slope aspect; VT, vegetation type; BG, β-1, 4-glucosidase; NAG, β-1, 4-N-acetylglucosaminidase; LAP, L-leucine aminopeptidase; ALP, alkaline phosphatase; CE, cellulase; PO, polyphenol oxidase; CA, catalase.

Our study showed that vegetation type (plantation, grassland, and primary forest), soil properties (SOC, TN, AN, TP, AP, BD, and SWC), microbial properties (MBC, MBN, MBP, and EEAs), and topography (elevation, slope gradient, slope aspect) explained approximately 89.45%, 54.71%, and 50.73% of the variations in GMEA, vector length, and vector angle, respectively (*p* < 0.01) (Figure 6). Among all driving factors, GMEA, vector length, and vector angle were primarily influenced by SOC, slope gradient, and ALP, respectively. The direct effects of vegetation type and topography on the variations in GMEA, vector length, and vector angle were less important, with relative contribution values of 5.45% and 17.84%, 4.25% and 17.01%, and 3.70% and 15.80%, respectively. Overall, GMEA and vector length were primarily influenced by soil properties, particularly chemical properties, while vector angle was predominantly affected by microbial properties, specifically the EEAs. The relative effects of EEAs on vector length and vector angle were approximately 33.22% and 39.74%, respectively. Among the individual enzymes, BG and ALP were the most important predictors of vector length and vector angle, respectively.

## 3. Discussion

### 3.1. Drivers of Soil EEAs and Microbial Nutrient Limitation

Shift in vegetation type alters EEAs by modifying the quantity and quality of soil resources available to microorganisms [32,33,34]. In this study, primary forest was characterized by relatively high soil nutrient and water availability, whereas EEAs were generally higher in plantation and grassland, which is consistent with the findings of Yan et al. [31]. Enzyme production represents a resource-acquisition strategy rather than simply a positive response to total nutrient availability [35]. When available resources are sufficient to meet microbial requirements, microorganisms may reduce their relative investment in enzyme production, whereas moderate resource limitation may stimulate greater enzymatic investment to acquire C, N, and P from organic substrates [27,36]. However, under severe resource depletion, limited C and energy availability may constrain microbial metabolism and enzyme synthesis, resulting in reduced enzyme production [37]. Accordingly, the lower enzyme investment in primary forest may reflect reduced demand under relatively high resource availability, whereas the higher enzyme investment in plantation and grassland may indicate resource conditions that stimulated enzyme production without severely constraining microbial metabolic capacity. In addition, plantation establishment on the Loess Plateau can increase not only total SOC but also the labile SOC fractions [38]. Such differences in substrate quality may explain why plantation exhibited higher EEAs than primary forest [31]. The highest EEAs observed in grassland may reflect resource conditions associated with natural secondary succession, which can alter plant inputs, microbial activity, and nutrient turnover, thereby influencing enzyme production [39]. Therefore, vegetation-mediated changes in substrate supply and nutrient cycling can regulate microbial enzyme investment during ecosystem recovery [40].

Among different environmental factors examined in our study, soil properties were strongly associated with variations in EEAs, with SOC emerging as an important predictor of overall enzyme activity. This finding is consistent with previous studies showing that increasing SOC can provide substantial substrates to microorganisms, thereby stimulating the synthesis of enzymes [41]. SOC may also contribute to the persistence of extracellular enzymes through the formation of humus–protein complexes [8,24]. Consequently, EEAs, especially N-acquiring enzyme activities, were positively correlated with SOC, which is consistent with the findings of Wu et al. [40]. N-acquiring enzymes participate in the depolymerization of organic N compounds and can therefore respond to both C supply and microbial N demand [42,43]. Our results further showed associations of EEAs with available nutrients, soil moisture, and bulk density, suggesting that enzyme allocation is jointly regulated by substrate availability, nutrient conditions, and physical constraints [44,45,46]. Topography was also associated with microbial resource limitation in our study, with slope gradient emerging as an important predictor of microbial C limitation [4]. Previous studies have shown that elevation and slope gradient were significantly associated with changes in EEAs through their effects on soil water availability, nutrient status, and vegetation distribution [46,47,48]. Our finding extends this relationship to microbial resource limitation, as steeper slopes may alter water retention, erosion, and the redistribution of organic matter and nutrients, thereby modifying the resource environment experienced by soil microorganisms [49].

Microbial nutrient limitation differed among vegetation types [50,51]. In our study, microbial C limitation was greater in plantation than in grassland and primary forest, which is consistent with the findings of Yan et al. [31]. Greater C limitation in plantation may indicate a higher microbial demand for metabolically available C relative to its supply [52]. In contrast, the lower C limitation in grassland may be related to the quantity and quality of C inputs [53]. Previous studies have shown that grass-derived inputs can contain relatively greater proportions of labile C and lower concentrations of structurally complex compounds such as lignin, potentially increasing C accessibility to microorganisms [40,54]. This mechanism provides a possible explanation for the lower microbial C limitation observed in grassland. Moreover, the observed relationships between microbial nutrient limitation and soil nutrient availability are consistent with previous studies showing that nutrient supply regulates the balance between microbial resource availability and demand [27,55], while soil moisture can further influence resource acquisition by regulating litter decomposition, substrate diffusion, and microbial activity [56]. These resource conditions may, in turn, affect microbial biomass and enzyme allocation. Consistent with this linkage, MBC was significantly associated with variation in microbial nutrient limitation, whereas the contributions of MBN and MBP were comparatively weak, which is consistent with the findings of Deng et al. [27]. At the enzymatic level, C-acquiring enzyme activity was strongly associated with microbial C limitation, whereas P-acquiring enzyme activity was closely associated with microbial N/P limitation, reflecting coordinated changes in enzyme allocation and microbial resource demand [3,57,58,59].

### 3.2. Predominant Microbial N Limitation Across Vegetation Types

Soil N and P are essential nutrients for plant growth and microbial metabolism, and imbalances in their availability can strongly constrain ecosystem functioning [60]. Previous studies have reported both N and P limitation of soil microbial metabolism following vegetation restoration on the Loess Plateau [40,61,62]. However, our study indicated that microbial metabolism was predominantly constrained by N rather than P. This finding is consistent with evidence that N availability represents an important constraint on vegetation recovery and microbial functioning on the Loess Plateau [63]. The Loess Plateau is characterized by relatively low precipitation and nutrient availability, which may constrain litter decomposition and nutrient turnover, thereby reducing the replenishment of plant and microbial AN [64,65]. Biological N inputs may also be comparatively limited in dryland ecosystems, resulting in a constrained N supply relative to ecosystem demand [66]. As vegetation develops following restoration, increasing plant biomass can further elevate ecosystem N demand and intensify competition for AN between plants and soil microorganisms [67]. This mechanism may be particularly relevant in plantations, where high stand biomass can increase nutrient demand even when N-fixing species are present [32]. For example, although the *Robinia pseudoacacia* plantation has the potential to increase N inputs through biological fixation, relatively high vegetation biomass may also increase plant N demand and thereby maintain competition with soil microorganisms for AN [62].

Our results are consistent with broader observations from dryland and afforested ecosystems in northern China. Dryland ecosystems at high northern latitudes are often susceptible to N limitation due to low precipitation and biological N inputs [68]. Nutrient imbalance and insufficient N availability have also been associated with the decline of shelterbelt forests in northern China [69]. Collectively, these findings suggest that increasing vegetation demand during restoration does not necessarily alleviate microbial N limitation and may instead intensify competition for a limited soil N pool [70]. Although N limitation predominated overall, some plantation and grassland plots exhibited potential microbial P limitation. Such spatial variability is also consistent with previous studies reporting increasing P limitation during vegetation restoration on the Loess Plateau [3]. Unlike N, soil P is primarily derived from parent material and rock weathering and can become progressively depleted as vegetation development increases biological P demand [40,71]. Increasing vegetation cover may further intensify plant–microbe competition for AP, while low soil moisture can restrict P diffusion and microbial access to available P pools [72]. These processes suggest that microbial nutrient limitation may shift from N toward P limitation as restoration proceeds on the Loess Plateau [3].

### 3.3. Microbial Strategies for Adapting to Environmental Constraints

Soil microbial biomass C:N:P stoichiometry is generally considered to be relatively homeostatic at the global scale [73]. However, recent studies have suggested that the strength of microbial stoichiometric homeostasis can vary among ecosystems and may change following vegetation restoration [9,40]. In this study, the relationships between microbial biomass stoichiometry and soil resource stoichiometry differed among vegetation types, indicating that microbial communities may adopt different strategies to cope with resource imbalance. In primary forest, microbial biomass stoichiometry showed greater adjustment in response to soil resource stoichiometry, although the significant negative slopes fell outside the predefined homeostasis classification framework. Primary forest is generally characterized by stable nutrient cycling and resource availability [13], and such stoichiometric adjustment may represent an alternative strategy for responding to variation in resource conditions rather than indicating weaker microbial functioning. Greater adjustment of microbial biomass composition may reduce the need for energetically costly resource-acquisition processes under some conditions, which could partly correspond with the lower enzyme investment observed in primary forest. Interactions between soil resource stoichiometry and microbial biomass composition can arise from nutrient transfer, compensatory regulation, and variation in microbial growth efficiency [74]. In particular, greater microbial growth efficiency under resources with lower C:N and C:P ratios may allow a larger proportion of assimilated C to be allocated to microbial biomass [75].

Microbial stoichiometric homeostasis also varied between plantation and grassland. In plantation, particularly *R. pseudoacacia*, microbial biomass stoichiometry showed comparatively greater plasticity, suggesting that microorganisms may partially adjust their biomass C:N:P in response to variation in soil resource stoichiometry. Such stoichiometric adjustment has been proposed as an adaptive mechanism that allows microbial communities to reduce elemental imbalance between biomass requirements and available resources [76]. Previous studies have suggested that changes in microbial community composition may contribute to this apparent plasticity because different microbial taxa can differ in biomass elemental composition and nutrient consumption strategies [77]. In addition, Bai et al. [63] highlighted that the fruit and branches of *R. pseudoacacia* have weak stoichiometric homeostasis, which may also affect the stoichiometric homeostasis of soil microbial biomass. In contrast, microbial biomass stoichiometry was comparatively more homeostatic in grassland. At the same time, we also found higher EEAs in grassland. Collectively, these findings suggested that microorganisms in grassland may have maintained relatively stable biomass elemental composition while increasing enzyme investment to acquire C, N, and P from soil resources. Therefore, strict stoichiometric homeostasis and greater enzyme investment may represent a coordinated resource-acquisition strategy that helps microorganisms maintain biomass elemental balance under variable resource conditions. Enzyme production can function as a compensatory resource-acquisition strategy when microbial biomass stoichiometry is strongly constrained [8,76]. Nevertheless, enzyme production itself requires substantial C and N investment, and excessive enzyme synthesis may become metabolically costly under strong nutrient limitation [78]. The relatively lower microbial N limitation observed in grassland may therefore have facilitated greater enzyme investment without imposing the same degree of nutrient constraint observed in plantation. Overall, strict homeostasis and weak homeostasis or stoichiometric plasticity should be considered different adaptive strategies adopted by microorganisms under contrasting environmental conditions, rather than indicators of superior or inferior microbial functioning.

### 3.4. Implications and Limitations

The contrasting patterns of SOC and EEAs observed among vegetation types may have important implications for understanding soil C recovery following vegetation restoration. Previous studies have shown that SOC concentrations in plantations remained below those of natural forests even after prolonged restoration [79]. Similarly, our study showed that primary forest was characterized by higher SOC, whereas restored vegetation generally exhibited higher EEAs. Greater enzymatic investment in restored vegetation may indicate more intensive microbial acquisition and turnover of organic C, potentially contributing to slower SOC accumulation [80]. In contrast, the lower EEAs observed in primary forest may reflect reduced microbial investment in resource acquisition under relatively resource-rich conditions and could be consistent with greater C retention [31]. Nevertheless, according to the soil microbial carbon pump framework, microbial processing can transform plant-derived C into microbial biomass and subsequently microbial necromass, which may become stabilized through association with soil minerals or physical protection within the soil matrix [81]. Thus, the higher EEAs observed in restored vegetation may enhance organic C decomposition and associated C losses, while simultaneously promoting the microbial transformation of labile C into more persistent forms. Future studies combining EEAs with measurements of microbial biomass and necromass, microbial carbon-use efficiency, particulate organic carbon, and mineral-associated organic carbon would help determine whether the greater microbial activity observed in plantation and grassland ultimately promotes SOC loss or stabilization.

In this study, the vegetation type was confounded with site characteristics, including location, elevation, plant species composition, topography, and land-use history. Therefore, differences among primary forest, plantation, and grassland cannot be attributed exclusively to vegetation type. In addition, the study represents a spatial comparison rather than a long-term chronosequence or manipulative restoration experiment, limiting inference about temporal trajectories and causal effects of vegetation restoration. Future studies using longer restoration chronosequences and nutrient-addition experiments are needed to clarify the temporal patterns and mechanisms of microbial nutrient limitation and determine whether shifts in nutrient limitation occur gradually or at particular stages of ecosystem recovery. Furthermore, the measured EEAs represent potential activities under standardized laboratory conditions and do not directly quantify in situ decomposition, nutrient mineralization, or C fluxes. Similarly, vector analysis provides proxies for microbial resource allocation and nutrient limitation rather than direct measurements of physiological nutrient limitation. Therefore, future studies incorporating long-term monitoring, nutrient-addition experiments, microbial community analyses, and microbial necromass biomarkers would be valuable for clarifying how alternative restoration pathways regulate microbial nutrient acquisition.

## 4. Materials and Methods

### 4.1. Study Sites

**Figure 7:**
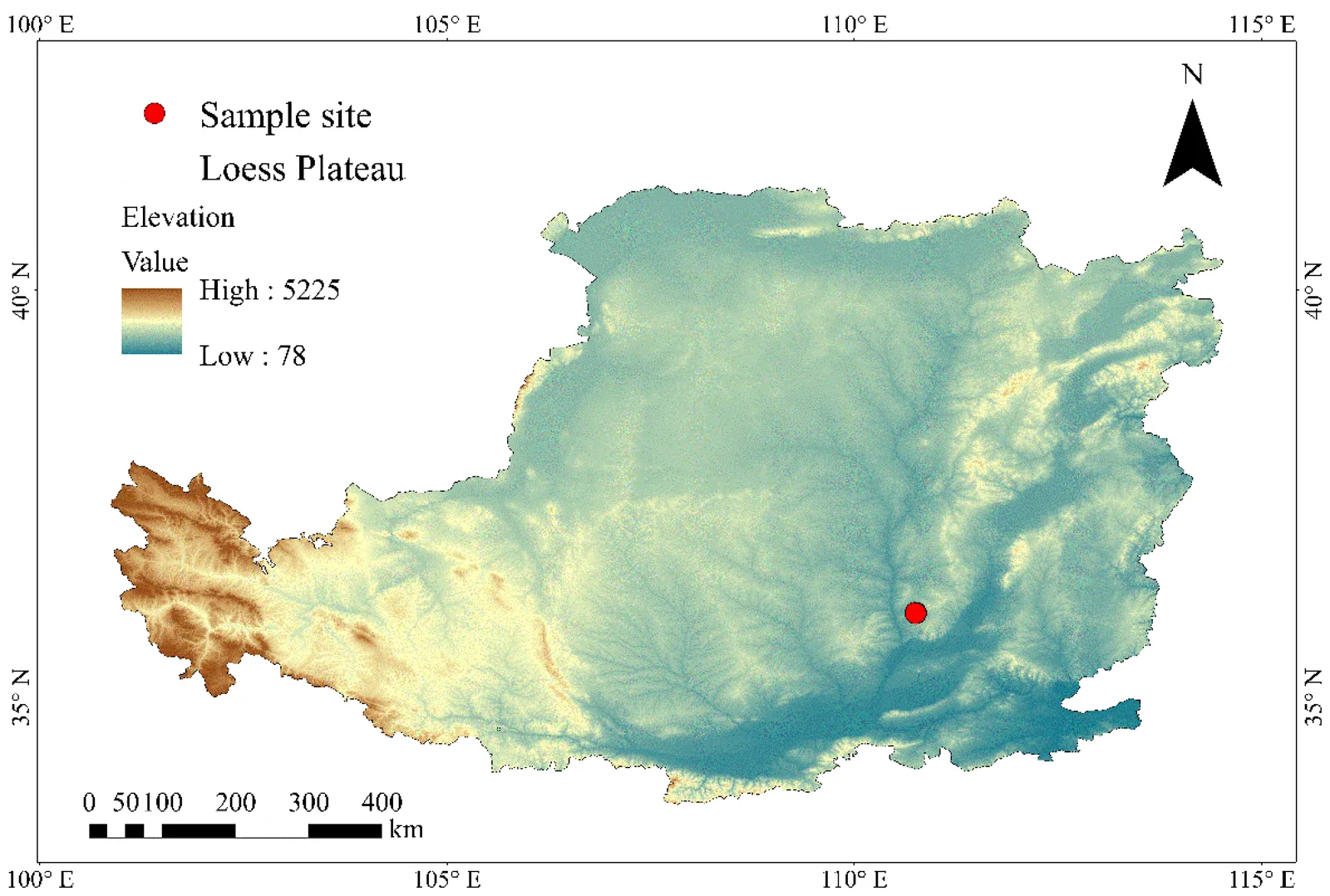
The location of sampling site on the Loess Plateau.

This study was carried out in the Caijiachuan Watershed, located in the hilly and gully region of the Loess Plateau, Ji County, Shanxi Province, China (36.23° N–36.3° N, 110.65° E–110.78° E) (Figure 7) [82]. The watershed is characterized by a semiarid continental monsoon climate, with an annual average air temperature of 10.2 °C, an annual frost-free period of 172 days, an annual average potential evapotranspiration of 1724 mm, and an annual average precipitation of 571 mm, which is unevenly distributed and mainly occurs from June to August [83]. The altitude ranges from 903 m to 1568 m, and the terrain is low in the west and high in the east. The main soil type is loess soil (Haplic Luvisol, FAO classification), which is derived from wind-deposited loessial parental materials and is susceptible to wind and water erosion. The main vegetation species are *Pinus tabuliformis*, *Populus* × *hopeiensis*, *Robinia pseudoacacia*, *Platycladus orientalis*, *Armeniaca sibirica*, *Quercus wutaishansea*, *Hippophae rhamnoides*, *Lespedeza bicolor*, *Bothriochloa ischaemum*, *Phragmites australis*, *Artemisia sacrorum*.

### 4.2. Soil Sampling

In July 2021, soil sampling was conducted in four vegetation types: an artificial *P. tabuliformis* plantation, an artificial *R. pseudoacacia* plantation, secondary grassland, and a *Quercus wutaishansea* primary forest. Specifically, *P. tabuliformis* and *R. pseudoacacia* plantations have the same stand ages (approximately 30 years), which have been confirmed by interviews with local farmers. Grassland is derived from secondary succession after cropland abandonment for approximately 30 years, while primary forest is conserved by the local government to prevent anthropogenic disturbance. For each vegetation type, three sampling sites were randomly selected. All sampling sites had similar climate conditions, and the distances among different sampling sites exceeded 15 m to eliminate the spatial autocorrelations of soil properties [7]. Detailed descriptions of the different sampling sites are recorded in Table 1. At each sampling site, three sampling plots with a size of 10 × 10 m^2^ were randomly set up in the middle of the slopes, and soil samples were collected after removing the ground litter layers. Five points were selected along an S-shaped pattern in each plot, and soil samples were taken from the 0–20 cm soil layers by a stainless steel tubular auger (inner diameter = 5 cm). Then, the five soil samples were thoroughly homogenized to form a composite sample, and three replicates were conducted. Therefore, a total of 36 sampling plots were established, and 108 soil samples were collected. All soil samples were sieved through a 2 mm screen to remove plant and stone debris and were divided into two subsamples: one subsample was air-dried to determine the soil physicochemical properties, while the other subsample was stored at 4 °C to determine the soil microbial biomass carbon (MBC), microbial biomass nitrogen (MBN), microbial biomass phosphorus (MBP), and EEAs [61].

EEAs are typically expressed as either absolute activity or specific activity (per unit of soil organic carbon) [26,80], both of which are considered early alerts that indicate slight variations in microbial activity and soil productivity [4,80]. Several studies have noted that absolute activity alone cannot discern whether observed changes in enzyme activities are attributed to differences in enzyme levels or are caused by soil carbon levels [26]. In contrast, specific activity can better indicate enzyme efficiency and microbial activities with different carbon levels [37,84].

### 4.3. Laboratory Analysis

Soil organic carbon (SOC) was determined by the Walkley-Black method [85]. Soil total nitrogen (TN) and available nitrogen (AN) were determined using an elemental analyzer [4]. Total phosphorus (TP) and available phosphorus (AP) were determined using a UV–Visible Spectrophotometer [4]. Soil pH was determined using a pH meter (PHSJ-4A pH meter, Zhangqiu Meihua International Trading Co., Jinan, China) in the 1:2.5 (w:v) suspension [27]. Soil bulk density (BD) was determined by dividing the soil oven-dried weight by the volume of the total core sample [3]. The soil water content (SWC) was measured gravimetrically by drying the soil samples in an oven at 105 °C for 48 h [31].

MBC, MBN, and MBP were determined by the chloroform fumigation extraction method [11]. In brief, 25 g of fresh soil was fumigated at 25 °C using ethanol-free CHCl_3_ or distilled water for 24 h. Then, the fumigated soil was extracted using 50 mL of K_2_SO_4_ and filtered [3]. Then MBC was determined using a TOC analyzer (TOCII, Elementar, Langenselbold, Germany); MBN was determined by ultraviolet spectrophotometric colorimetry; and MBP was determined by the molybdenum-antimony spectrophotometric colorimetry method [31]. The experimentally derived conversion factors of MBC, MBN, and MBP were 0.38, 0.54, and 0.4, respectively [32]. EEAs were assayed using 3 g of fresh soil that was incubated at 37 °C for 24 h with the appropriate substrates. Specifically, BG, NAG, LAP, ALP were measured following modified versions of standard fluorometric techniques, which have been fully described in Peng and Wang [9]. The determination of PO and CA has been fully described in Chen et al. [4]. In addition, CE was determined with 1% carboxymethyl cellulose solution and phosphate buffer. The suspension was treated with 3,5-dinitrosalicylic acid, and the activity was assayed in an ultraviolet spectrometer subsystem at 540 nm [80].

In our study, we also calculated the geometric mean of enzyme activity (GMEA) to represent the average enzyme activity levels of different vegetation types. The equation to determine the GMEA was as follows [11]:

(1) $$\mathrm{GMEA}=\sqrt[{6}]{\mathrm{BG}\times (\mathrm{NAG}+\mathrm{LAP})\times \mathrm{ALP}\times \mathrm{CE}\times \mathrm{PO}\times \mathrm{CA}}$$

where BG, NAG, LAP, ALP, CE, PO, and CA represent β-1, 4-glucosidase, β-1, 4-N-acetylglucosaminidase, L-leucine aminopeptidase, alkaline phosphatase, cellulase, polyphenol oxidase, and catalase, respectively.

### 4.4. Determination of Microbial Nutrient Limitation

Given that ecoenzymatic stoichiometry can reflect the biogeochemical equilibrium between nutrient availability and microbial demand, it has been considered an important tool for predicting microbial nutrient limitations in various ecosystems [27,86,87]. BG, (NAG + LAP), and ALP exhibit identical magnitudes and are typically associated with biogeochemical processes and microbial metabolism within soil [4,28]. Therefore, the ecoenzymatic stoichiometry of BG (C- degrading enzymes) vs. ALP (P-acquiring enzymes) and BG vs. (NAG + LAP) (N-acquiring enzymes) has been considered an indicator of microbial nutrient limitations [28,57]. Prior to calculating ecoenzymatic stoichiometry, EEAs were normalized to specific enzyme activity (μg product g^−1^ SOC d^−1^) by dividing absolute enzyme activity by SOC [11]. Microbial nutrient limitations were assessed by vector analysis, and the equations to calculate vector length and vector angle were as follows [11,88]:

(2) $$\mathrm{Vector}\;\mathrm{length}=\;\sqrt{\left(X^{2}+Y^{2}\right)}$$

*Vector angle* = *Degrees*[*ATAN*2(*X*,*Y*)] (3)

where X is the relative activity of C- vs. P-acquiring enzymes (e.g., ln(BG)/ln(ALP)), and Y is the relative activity of C- vs. N-acquiring enzymes (e.g., ln(BG)/ln(NAG + LAP)). A longer vector length represents a greater C limitation, while vector angles < 45° and >45° represent the relative degrees of N and P limitations, respectively [11,88].

### 4.5. Microbial Biomass Stoichiometric Homeostasis

The stoichiometric homeostasis of microorganisms is considered the degree to which microorganisms maintain a stable C:N:P ratio despite changing chemical composition and availability of their resources [89]. In particular, microbial biomass stoichiometry is considered strictly homeostatic when it remains unchanged in response to variations in soil nutrient stoichiometry [3]. Therefore, the equation to determine the degrees of community-level microbial C:N and C:P homeostasis (H) was as follows [11,25]:

(4) $$H=\;\frac{\log_{10}(x)}{\log_{10}\left(y\right)-\log_{10}(c)}$$

where x is the resource nutrient stoichiometry (e.g., soil C:N or C:P), y is the microbial biomass nutrient stoichiometry (e.g., microbial biomass C:N or C:P), and c is a constant [11]. The value 1/H is the slope of the regression of log10(y) on log10(x). H ≫ 1 indicates strict homeostasis, whereas H ≈ 1 indicates weak or no homeostasis. For significant regressions with 0 < 1/H < 1, homeostasis was classified as homeostatic (0 < 1/H < 0.25), weakly homeostatic (0.25 < 1/H < 0.5), weakly plastic (0.5 < 1/H < 0.75), or plastic (0.75 < 1/H < 1). Nonsignificant regressions (*p* > 0.05) were classified as strictly homeostatic [25,89]. When a regression was significant but the 1/H slope was negative, the slope was considered to fall outside the predefined positive-slope classification framework and was therefore not assigned a homeostasis class.

### 4.6. Statistical Analyses

The statistical analyses were conducted using IBM SPSS statistics 26.0 (SPSS Inc., Chicago, IL, USA) and R 4.1.2 (Project for Statistical Computing, Vienna, Austria). Differences in EEAs among vegetation types were determined using one-way analysis of variance (ANOVA). The normality and homogeneity of variance of all variables were checked before analysis. When a significant effect of vegetation type was detected, pairwise differences among vegetation types were determined using the least significant difference (LSD) at the 0.05 significance level. Principal component analysis (PCA) was performed to indicate the variations in soil properties among different vegetation types using the *factoextra* package in R. The correlations between EEAs and soil properties were determined based on the absolute EEAs and were conducted via Pearson’s test using the *Corrplot* package in R. The relative importance of different influencing factors on GMEA, vector length, and vector angle was determined by the random forest algorithm using the *randomForest* and *rfPermute* packages in R [90]. The importance of each influencing factor was evaluated by observing the increase in the mean squared error (%IncMSE), and the parameters ntree, mtry, and nrep were set to 500, 3, and 1000 in each test, respectively. Before the random forest analysis was conducted, different vegetation types and slope aspects were represented by numerically coded ordinal variables: 0 = plantation, 1 = grassland, 2 = primary forest; 0 = shady slope, 1 = sunny slope [4]. In addition, the specific enzyme activities, vector length, vector angle, microbial community homeostasis, and the results of the PCA and random forest algorithm were all presented using Origin Pro 8.0.

## 5. Conclusions

On the Loess Plateau, primary forest was characterized by relatively high soil nutrient and water availability. In contrast, restored vegetation, including plantation and grassland, generally exhibited higher EEAs. Ecoenzymatic stoichiometry indicated greater microbial C limitation in plantation than in grassland and primary forest. Microbial nutrient limitation was predominantly characterized by N rather than P limitation across vegetation types. Variations in EEAs and microbial nutrient limitation were associated with multiple environmental factors. Specifically, SOC was an important predictor of EEAs, whereas slope gradient was associated with microbial C limitation. ALP was strongly associated with microbial N/P limitation. Microbial biomass stoichiometric homeostasis also differed among vegetation types, with greater stoichiometric adjustment in plantation and strict homeostasis in grassland. Overall, our study reveals contrasting microbial resource-acquisition and stoichiometric strategies among primary forest, plantation, and grassland and highlights the importance of considering both soil resource conditions and microbial enzyme allocation when evaluating vegetation restoration. The comparatively high enzyme investment and lower microbial limitation observed in grassland suggest that natural secondary succession following cropland abandonment may support favorable soil microbial functioning.

## Figures and Tables

**Table 1:** General information on the sampling sites.

Vegetation Type	Plant Community	Geographic Coordinates	Stand Height (m)	Stand Diameter (cm)	Canopy Cover (%)	Elevation (m)	Slope Aspect	Slope Gradient (°)
Plantation	*Pinus tabuliformis*	36.039° N 110.759° E	11.53	14.46	50	1361.33	Shady	29
		36.040° N 110.756° E	10.46	13.98	48	1360.27	Sunny	18
		36.044° N 110.752° E	11.57	13.74	42	1303.43	Sunny	9
	*Robinia pseudoacacia*	36.040° N 110.761° E	10.45	13.21	69	1358.29	Sunny	20
		36.041° N 110.757° E	10.95	12.60	72	1341.88	Sunny	15
		36.044° N 110.752° E	11.22	12.97	70	1339.76	Shady	15
Grassland	*Artemisia gmelinii*	36.037° N 110.763° E	-	-	-	1286.21	Shady	20
		36.034° N 110.755° E	-	-	-	1241.62	Shady	24
		36.030° N 110.750° E	-	-	-	1240.77	Sunny	11
Primary forest	*Quercus wutaishansea*	36.259° N 110.647° E	12.16	13.70	67	1624.31	Shady	17
		36.260° N 110.633° E	12.20	13.17	61	1612.14	Sunny	31
		36.268° N 110.640° E	12.82	14.02	68	1600.75	Shady	30

## Data Availability

The data presented in this study are available from the corresponding author upon reasonable request.

## Abbreviations

The following abbreviations are used in this manuscript:

SOC	Soil organic carbon
TN	Total nitrogen
TP	Total phosphorus
BD	Bulk density
SWC	Soil water content
AN	Available nitrogen
AP	Available phosphorus
MBC	Microbial biomass carbon
MBN	Microbial biomass nitrogen
MBP	Microbial biomass phosphorus
BG	β-1,4-glucosidase
NAG	β-1,4-N-acetylglucosaminidase
LAP	L-leucine aminopeptidase
ALP	Alkaline phosphatase
CE	Cellulase
PO	Polyphenol oxidase
CA	Catalase
EEAs	Extracellular enzyme activities
GMEA	Geometric mean of enzyme activity
